# Integrating Performance Records and Genetic Evaluations in Spanish Horse Populations Competing in Olympic Disciplines

**DOI:** 10.3390/life16030455

**Published:** 2026-03-10

**Authors:** María Dolores Gómez, María José Sánchez-Guerrero, Davinia Isabel Perdomo-González, María Ripollés-Lobo, Ester Bartolomé, Mercedes Valera

**Affiliations:** 1 Departamento de Agronomía, Escuela Técnica Superior de Ingeniería Agronómica, Universidad de Sevilla, Ctra. Utrera Km 1, 41013 Sevilla, Spain; msanchez73@us.es (M.J.S.-G.); mripolles@us.es (M.R.-L.); ebartolome@us.es (E.B.); mvalera@us.es (M.V.); 2 Departamento de Producción Animal, Facultad de Veterinaria, Universidad Complutense de Madrid, Avda. Puerta de Hierro s/n, 28040 Madrid, Spain; daperdom@ucm.es

**Keywords:** BLUP genetic evaluation, breeding program evaluation, selection intensity, sport horse performance

## Abstract

This study evaluates performance data and genetic merit of the main horse populations competing in Olympic disciplines in Spain and examines their implications for the optimization of official Breeding Programs. Performance records from 2004–2023 were analyzed, including 101,093 participations in Dressage, 319,000 in Show Jumping, and 17,535 in Eventing. These records were combined with pedigree information from 35,589 horses in Dressage, 33,935 in Show Jumping, and 12,102 in Eventing and evaluated using BLUP animal models to obtain standardized Estimated Breeding Values (EBV; mean 100 ± 20) and a Genetic Global Index (GGI). A single unified evaluation model was implemented for all studbooks, enabling a direct comparison of genetic quality across different breeds. Results revealed marked differences in genetic merit and genetic progress among breeds. Similar mean EBVs were obtained for the three analyzed breeds in Dressage in both the complete and the top 10% populations, with positive genetic trends in Caballo de Deporte Español (CDE) and Pura Raza Española (PRE), while the slope of EBV on birth year was not significantly different from zero in Spanish Anglo-Árabe (AA). CDE showed the highest mean EBVs and accuracies in Show Jumping (EBV up to 109.27; R up to 0.72), with a clear positive genetic trend. In Eventing, CDE and AA showed similar EBVs, while PRE consistently exhibited lower ones, although with a comparatively more favorable genetic trend. Analysis of selection intensity indicated that PRE breeders applied the most consistent genetic criteria, preferentially using animals with GGI > 100, whereas CDE and AA showed discrepancies between genetic merit and reproductive use. Overall, the unified Spanish genetic evaluation system provides reliable comparative information across breeds and has enabled measurable genetic progress, although improvements in breeders’ decision-making and in the use of genetic information are needed to maximize selection response.

## 1. Introduction

Sport horses are bred worldwide with the aim of competing in the Olympic disciplines of Show Jumping, Dressage, and Eventing [1]. In horses, as in human athletes, performance in a discipline results from the convergence of several favorable factors. Intrinsic factors include neuro-sensory qualities, cardio-respiratory capacity, muscular energy potential, and locomotor characteristics. These factors are partly innate, then developed and influenced by extrinsic factors such as breeding conditions, training, and equine management [2]. Because of this multifactorial nature, breeding strategies aimed at improving sport performance must consider both the biological determinants of athletic ability and the structure of the competitive environment in which performance is expressed.

The success of the sport horse industry is multifactorial; however, implementing successful Breeding Programs and achieving genetic improvement are vital to maintaining this success. Unlike environmental effects such as feeding or management, genetic gain is cumulative and permanent [3], making selection a central tool for long-term progress. The primary objective of Breeding Programs for sport horses is to identify and select genetically superior animals capable of performing successfully at the highest competitive levels [4]. In this context, genetic evaluations provide the quantitative basis for selection decisions by estimating the breeding values for performance-related traits, thereby enabling breeders to make informed mating decisions.

Many sport horse studbooks already have Breeding Programs in place to identify and select genetically superior stallions and mares to become parents of the next generation [5]. However, the estimated breeding values (EBVs) published and the traits included in these evaluations vary among studbooks. This variability may be attributed to differences in the breeding goals between studbooks or simply to the availability and heterogeneity of data [6].

In most European countries, performance testing of sport horses in competitions has been carried out in a standardized way over many years, enabling genetic analyses based on large datasets comprising thousands of competition records [7], since data from horses participating in competitions are directly related to the breeding goals established for each breed. Moreover, information collected early in life during the studbook inspections and performance tests can be used for genetic evaluation, as these traits are genetically correlated with the breeding objectives [8]. This combination of early-life data and competition results strengthens the accuracy and relevance of genetic evaluations for sporting performance.

Horse breeding has a long tradition in Spain, and the Olympic disciplines form part of the breeding objectives of several national studbooks. In this context, the main Spanish studbooks involved in Olympic disciplines, including those representing both sport-oriented and traditionally versatile breeds, operate under a unified national evaluation framework. Competition data for Dressage, Show Jumping, and Eventing have also been collected by the Royal Spanish Equestrian Federation and provided to the different studbooks for the genetic evaluation of their breeding stock. Furthermore, since 2004, performance tests for young horses have been organized to collect early information on the animals for inclusion in the official Breeding Programs, thereby facilitating earlier genetic evaluations and reducing the generation interval. Both types of data are combined to develop a single annual breeding evaluation by discipline. Each breed publishes its data in an official Breeding Stock Catalogue, offering objective and complete information to breeders for mating decisions and making results comparable between breeds. This integrated system constitutes a distinctive national model within Europe, as it combines multiple data sources across several studbooks under a common evaluation structure. Despite the relevance of this system, no comprehensive description has yet been published that integrates the available performance information with the structure and outcomes of the national genetic evaluation framework. The main aim of this publication is to analyze the performance information available for the Olympic equine disciplines in Spain and to examine the contribution of the national genetic evaluation system to the observed genetic progress of the main equine populations that include these disciplines in their official Breeding Programs. By addressing this gap, the study provides an evidence-based overview of the Spanish system and its capacity to support long-term selection for sport performance, offering insights potentially applicable to other European breeding schemes.

## 2. Materials and Methods

### 2.1. Performance Data

Annual performance data for all horses participating in Young Horse Tests and official competitions held in Spain between 2004 and 2023 for the Olympic disciplines of Dressage, Show Jumping, and Eventing were used in this study. These data are systematically used in the annual breeding evaluation of the animals in Spain. The Young Horse Tests are specific performance controls for young horses, from age 4 to 7 years, held by the Breeders’ Associations to obtain objective performance data to be included in the official Breeding Programs. Official competition data are collected from the dataset of the Royal Spanish Equestrian Federation at the end of each year (https://rfhe.com/), whereas data on the Young Horse Tests are supplied by the Breeders’ Associations that organize these performance controls. For each horse participating in these performance evaluations, identification data (name, Universal Equine Life Number, birth date, breed, sex, and microchip number) and performance records (points or penalties for each exercise and trait) were available. Additionally, information on systematic environmental effects that may influence the obtained results, such as rider, date, location/event, and competition level, was also included in the dataset. For Eventing, only horses completing the three phases of the discipline (Dressage, Show Jumping, and Cross Country) were retained to ensure comparability of phenotypes and to avoid elimination-related bias, as the genetic evaluation treats the three phases as correlated traits within a unified evaluation framework. Table 1 provides a description of the material available for each breed and Olympic discipline in Spain, including information about the total number of participations and participating horses, grouped by sex.

### 2.2. Genetic Models

Available performance data have been systematically used in the annual genetic evaluation of the animals bred in Spain, grouped by discipline. For the genetic evaluation and estimation of EBVs, a Best Linear Unbiased Prediction (BLUP) animal model was used. Specifically, variance components were estimated using a Bayesian approach via Gibbs sampling implemented in the GIBBSF90+ module of the BLUPF90 software package version 3.16 [9]. Posterior means and standard deviations were obtained using POSTGIBBSF90 to derive estimates of additive genetic variance and heritability. The models applied to the genetic evaluation for each discipline, including analyzed traits, estimated heritability values, weighted Genetic Global Index (GGI), methodology, genetic model structure, and total number of animals included in the pedigree file, are described in Table 2, taking as reference the genetic evaluation implemented in 2024 (performance data between 2004 and 2023). A total of six, two, and three traits were evaluated for Dressage, Show Jumping, and Eventing, respectively. For the publication in the Breeding Stock Catalogues, the EBVs were transformed to a scale with a mean of 100 and a standard deviation of 20. They are also included in a GGI, which weights the different traits evaluated according to their importance in the genetic selection and the breeding objectives of the indicated breeds. The heritability values obtained for the analyzed traits ranged between 0.04 (PSJ) and 0.19 (EDr) and were consistent with previously published estimates [4,10,11,12,13]. These results confirm that the evaluated performance traits present sufficient additive genetic variance to be improved through selection.

### 2.3. Studbook and Official Breeding Program Information

Due to national and European regulations, including EU zootechnical legislation, all horse breeds in Spain are required to have an officially approved Breeding Program for the selection and/or conservation of genetic resources, which regulates studbook and breeding conditions. Table 3 summarizes the main information on the horse breeds that systematically participate in official competitions and performance tests held in Spain, and the orientation of their official Breeding Programs. This table provides the structural framework necessary to interpret the comparative genetic analyses presented below.

For each horse with individual performance records, up to three generations of ancestors were included, if available, in the pedigree file for the genetic evaluation. Pedigree data were obtained from the national studbook of each breed, which contains records for all horses with a passport and registered pedigree data. Pedigree data from foreign stallions and breeding mares are also included. The evaluations include a total of 35,589, 33,935, and 12,102 animals in Dressage, Show Jumping, and Eventing disciplines, respectively (Table 2).

### 2.4. Genetic Progress and Selection Intensity

For the genetic analysis of the evolution of the EBVs for the Olympic equestrian disciplines in Spain, only the three populations that incorporate these disciplines into their official Breeding Programs and that recorded more than 5000 participations in at least one Olympic discipline during the study period were included: Pura Raza Española (PRE), Caballo de Deporte Español (CDE) and Spanish Anglo-Árabe (AA), since genetic efforts in these breeds are specifically aimed at improving performance in these disciplines. The remaining populations have breeding objectives that are not primarily oriented towards Olympic performance, as shown in Table 3.

As the breeding evaluation is carried out jointly for the entire population of animals under performance testing in Spain, the EBVs (standardized to a scale of 100 ± 20) can be used to compare the genetic quality of the animals across different breeds because a single unified evaluation model is implemented across all studbooks. In addition, overall genetic quality can be assessed using the GGI, which weights the different EBVs according to the relative importance percentages shown in Table 2.

To quantitatively estimate genetic trends and selection response for each discipline and breed, a simple linear regression analysis was performed using Microsoft Excel^®^ (2021). The average Estimated Breeding Values (EBV) per birth year were defined as the dependent variable (y), while the animals’ birth year (period 1999–2019) served as the independent variable (x). Annual genetic progress was estimated through the regression coefficient (β), reported together with its standard error (s.e.) to indicate the precision of the estimate, representing the annual change in EBV units. Model goodness-of-fit was evaluated using the coefficient of determination (R^2^), and the statistical significance of the trends was assessed via *p*-values. Significance levels were set at *p* < 0.05 for significant trends and 0.05 < *p* < 0.10 for marginal trends.

The genetic quality of each breed within the national evaluation framework for the three Olympic disciplines is estimated based on the average EBVs of the most relevant traits for each discipline and the GGI. The reference population for this analysis includes only animals born in or after the year 2000 with an average GGI value above 100, also taking into account animals belonging to the top 10% ranked by GGI.

The evaluation of the genetic progress is based on the analysis of the evolution of the EBVs for the GGI, grouped by breed and birth year, for all horses included in the study registered in the different sections of the PRE, CDE and AA studbooks, grouped by Olympic discipline and by comparing the EBVs between the complete population and the elite group (top 10%).

Finally, to assess selection intensity within these breeds, the average number of offspring per stallion or mare was estimated according to their GGI value and the age class of the breeding stock. Only registered offspring were considered, ensuring consistency across studbooks. This approach allows the evaluation of potential variations in the number of registered offspring in relation to the GGI value of the parents.

## 3. Results

Table 1 summarizes the structure of the available performance data for each Olympic discipline and breed. The highest average number of participations per event was recorded in Show Jumping (160.14; SD = 158.70), followed by Dressage (68.91; SD = 62.47) and Eventing (27.40; SD = 21.17). Participations correspond to individual starts; therefore, the same horse may contribute multiple starts within the same event or across different events.

In Dressage competitions held in Spain, PRE showed the highest number of participations (43,921) and individual competitors (4563), followed by Foreign Warmblood Horses—FWH—(39,503 participations and 2723 individuals), with males being the most represented sex category. In Show Jumping, FWH horses accounted for the majority of participations (202,915) and competitors (6772), most of them males, followed by CDE horses, with 89,091 participations from 3422 individuals, the majority being females. In Eventing, CDE was the most represented breed (5296 participations and 568 individuals), followed by AA (5109 participations and 443 individuals). Although the number of CDE male competitors was higher (259 vs. 243 females), most participations corresponded to CDE females (2393 vs. 1943 for males).

Overall, considering individual animals, most competing horses were males across the three Olympic disciplines analyzed (6348 in Dressage, 5389 in Show Jumping, and 839 in Eventing). Notably, in Dressage, geldings outnumbered females in six of the nine populations considered (Spanish Pura Raza Árabe -PRA-, Pura Sangre Inglés -PSI-, Spanish Hispano-Árabe -HA-, FWH, Pony breeds -Pon- and Others -Oth-); whereas the number of females was considerably higher than geldings in the PRE population (326 female vs. 126 gelding). In Show Jumping, the number of CDE females (1606) exceeded that of males (1413) and geldings (403). Finally, in Eventing, the number of females surpassed that of males in five of the nine breed groups: HA, AA, FWH, Pon and Oth.

Table 2 presents a description of the model used for the genetic evaluation of each Olympic discipline in Spain. Notably, the number of animals included in the pedigree file for Dressage (35,589) is higher than in the other disciplines (33,935 in Show Jumping and 12,102 in Eventing), despite the lower number of registered participations (101,093) and participating horses (9073) compared to Show Jumping (319,000 and 12,109, respectively) (Table 1). This pattern may reflect the greater pedigree depth available for the national breeds (mainly PRE), which dominate Dressage competitions, whereas Show Jumping and Eventing include a higher proportion of foreign-origin horses with fewer known/registered generations in the official studbooks.

Heritability estimates for all analyzed traits are also shown in this table, ranging from 0.10 (TP) to 0.18 (T, C and P) in Dressage, 0.04 (PSJ) to 0.10 (WFR) in Show Jumping, and 0.09 (ECr) to 0.19 (EDr) in Eventing.

Table 3 summarizes the main characteristics of the most numerous horse breeds bred in Spain and the focus of their official Breeding Programs. These programs cover different disciplines, and although performance data are available for most breeds and genetic evaluations are conducted, the majority of PRE horses genetically evaluated in 2024 obtained EBVs for Dressage (43,914 individuals). Nevertheless, a considerable number of PRE horses were also evaluated for Eventing ability (2310). CDE horses were primarily evaluated for Dressage (5942), as were HA individuals (188). However, a substantial number of CDE horses were also evaluated for Show Jumping (4747). The number of PRA and AA horses genetically evaluated for Eventing was also noteworthy (1490 and 1485, respectively). Finally, PSI horses, which are mainly used in speed competitions in Spanish racetracks, were primarily evaluated for Show Jumping (2154) when considering only the Olympic disciplines.

**Figure 1:**
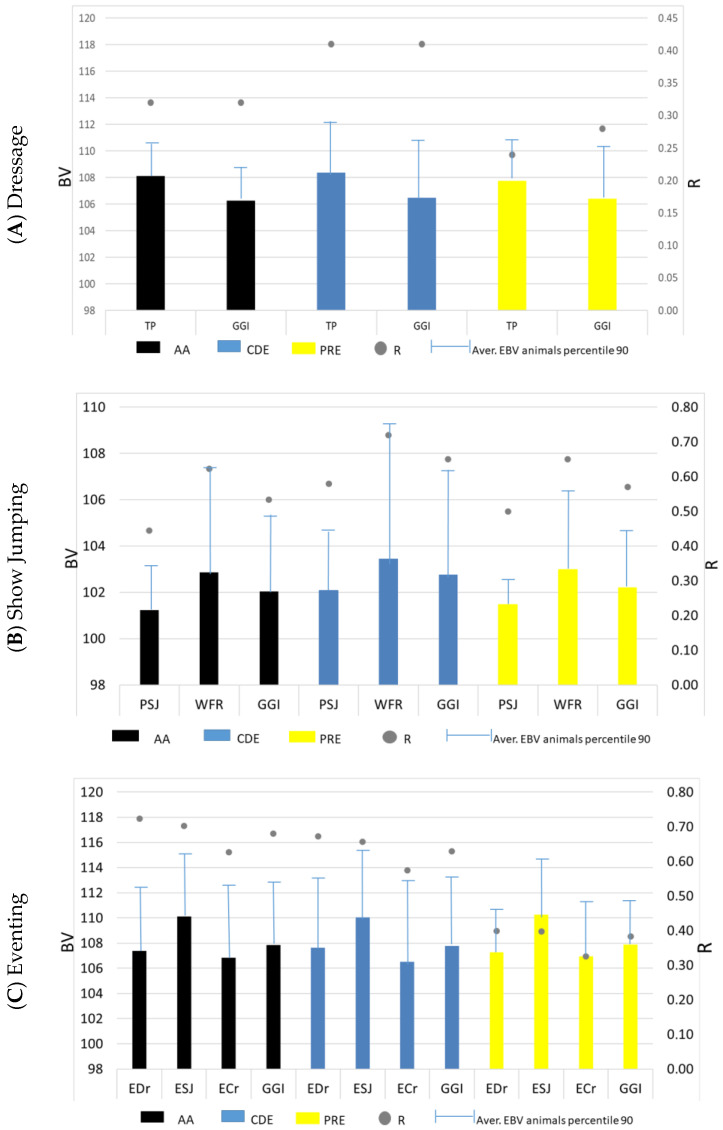
Average EBVs, top 10% EBVs and accuracy (R) for the main traits and the Genetic Global Index (GGI) across the three Olympic disciplines, by breed. EBV = Estimated Breeding Value; TP = total points per reprise; PSJ = positive score of Show Jumping; WFR = weighted final ranking; EDr = positive score of Dressage phase in Eventing; ESJ = positive score of Show Jumping phase in Eventing; ECr = positive score of Cross-country phase in Eventing; GGI = Genetic Global Index; R = accuracy. Only Pura Raza Española (PRE), Caballo de Deporte Español (CDE) and Spanish Anglo-Árabe (AA) were included, as these are the populations whose official Breeding Programs incorporate Olympic disciplines.

The comparison of genetic merit among the three populations genetically evaluated for each Olympic discipline in Spain (AA, CDE and PRE) is shown in Figure 1. This figure displays the average EBVs and accuracies (R) by discipline and breed, as well as the average EBV of animals within the top 10% percentile.

For Classic Dressage, only the total points/reprise (TP) and the Genetic Global Index (GGI) are represented (Figure 1A), which summarize overall performance ability. The average values for TP and GGI were consistently above 100, which corresponds to the mean of the evaluated population. When the GGI is considered, very similar results were observed for the different analyzed populations, with values ranging between 106.28 for AA horses and 106.50 for CDE. TP showed the highest EBV in all the analyzed populations, ranging between 107.75 for PRE and 108.38 for CDE. The highest R values were obtained for the CDE population, and the lowest for the PRE.

In Show Jumping, all the estimated traits are represented: positive score of jumping (PSJ), weighted final ranking (WFR) and GGI (Figure 1B). All populations showed EBVs equal to or higher than 100, the average of the population, for the three represented traits. Higher EBV and R values were observed in the CDE animals, followed by the PRE, with only minor differences compared to AA horses.

Finally, in Figure 1C for the Eventing, all the analyzed traits are included: positive score of dressage (EDr), positive score of jumping (ESJ), positive score of cross-country (ECr) and GGI. All populations showed EBVs equal to or higher than the population mean of 100 in all traits. The average GGI value is similar for all the represented populations (107.86 for AA horses, 107.80 for CDE and 107.89 for PRE horses). Overall, all populations showed their highest EBVs in the jumping-related traits, while the lowest EBVs were consistently observed for the cross-country trait. The highest R values were obtained for the AA animals, followed by the CDE, whereas PRE horses showed the lowest accuracies.

**Figure 2:**
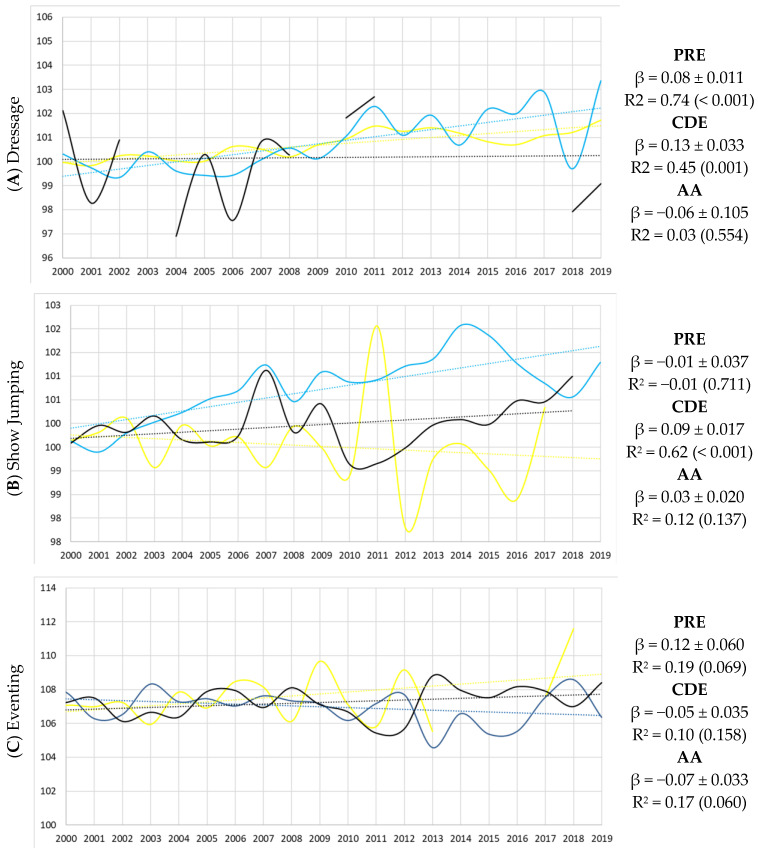
Genetic progress by Olympic discipline and breed (Pura Raza Española (PRE) = yellow, Caballo de Deporte Español (CDE) = blue and Spanish Anglo-Árabe (AA) = black) based on the Genetic Global Index (GGI) for the three populations whose official Breeding Programs include these disciplines. GGI = Genetic Global Index; PRE = Pura Raza Española; CDE = Caballo de Deporte Español; AA = Spanish Anglo-Árabe. β (EBV/year) represents the slope of the trend line, with its standard error indicating the magnitude and direction of the trend; R^2^ is the coefficient of determination which indicates the proportion of variance explained by the model and *p*-value is shown in brackets. Evolution is included as a continuous line and tendency as a dashed line. Note: The Spanish Anglo-Árabe curve is discontinuous because several birth years contained no AA horses with sufficient pedigree depth and/or performance records to obtain a Dressage EBV. The figure therefore reflects the actual availability of data by cohort.

The genetic progress of the three main breeds that incorporate the Olympic disciplines into their official Breeding Programs was assessed by examining the temporal trend of the GGI values by year of birth of the animals included in the analysis. The graphical representation and the results of the linear regression analyses are shown in Figure 2.

For Dressage, the temporal evolution of the GGI indicates a slight, but significant, upward trend in PRE (β = 0.08 ± 0.011) and CDE (β = 0.13 ± 0.033). A modest decreasing tendency was observed in AA. However, the interpretation for AA should be made cautiously due to the limited number of animals contributing to the Dressage evaluation for this breed, as evidenced by their discontinuous curve and the lack of statistical significance.

The temporal evolution of the GGI for Show Jumping indicates a pronounced and significant upward trend in CDE (β = 0.09 ± 0.017), whereas no significant trends were observed in PRE and AA. This pattern is consistent with the breeding orientation of the CDE population, primarily selected for Show Jumping performance. In contrast, the evolution of GGI for Eventing is less marked, with a slight increase observed in PRE (β = 0.11 ± 0.060) and overall stability in AA (β = −0.07 ± 0.033), both with marginal significance.

Table 4 summarizes the mean EBV, the EBV of the top 10% of animals and the accuracy values for the main traits evaluated in Dressage, Show Jumping and Eventing for PRE, CDE and AA populations. In Dressage, the three populations showed similar EBV levels for both TP and GGI, whereas CDE and PRE maintained competitive figures within the top-ranked group, as evidenced by the average EBV for top 10% animals in comparison with AA horses. In Show Jumping, CDE obtained the highest EBVs across PSJ, WFR and GGI, reflecting a stronger specialization for this discipline compared to PRE and AA, both in the complete and the top 10% populations. In Eventing, CDE and AA again showed superior EBV levels for EDr and GGI, while similar values were shown for ESJ and ECr. PRE displayed lower averages but clear improvement within the top 10% of animals in the four analyzed traits.

Overall, the results indicate marked differences in genetic merit among breeds, with CDE showing the highest EBVs in Show Jumping and Eventing, AA showing consistent competitiveness across disciplines, and PRE presenting more moderate but still positive values, particularly among its top 10% ranking individuals.

In Table 5, as an indicator of the selection intensity, the average number of offspring per stallion or mare according to their GGI value and the age class of the breeding stock was considered to evaluate potential variations in the number of registered offspring in relation to the GGI value of the parents.

In general, younger animals considered young horses in the official competitions (≤7 years) show a lower number of registered offspring compared to older stallions and broodmares, with this trend being more pronounced in mares. Although there are some PRE stallions with registered offspring in this age group for Dressage, with an average between 1.15 and 1.62 registered descendants, the highest number of registered offspring is observed in animals (stallions and mares) included in the top 10% by GGI value and older than twelve years. However, the selection criteria for breeding stock appear unclear, as the animals with the highest number of registered offspring do not always coincide with those included within the top 10% of GGI across all Olympic disciplines and sexes.

## 4. Discussion

Many horse breeding organizations aim at improving the genetic ability of riding horses for performance in sport competitions, mainly in Dressage, Show Jumping and Eventing, the Olympic equestrian disciplines [1]. Within this context, national genetic evaluation systems play a key role in translating performance records into effective selection decisions.

The inclusion of molecular data (SNPs) could improve the accuracy of the genetic evaluations. However, genomic information is not available for all animals included in performance recording over the last 20 years, as it has not yet been implemented across all equine populations bred in Spain, and the pedigree-based BLUP methodology is the official system used for routine genetic evaluation. Therefore, it remains the only methodology currently available for conducting a longitudinal analysis of genetic merit and genetic progress in Spanish horse populations. Although future research within the Spanish national breeding framework could incorporate genomic evaluation (e.g., ssGBLUP), as well as functional longevity and structural conformation traits (including axial and appendicular musculoskeletal soundness), these components are not yet implemented uniformly at the national level and were therefore outside the scope of this study.

As shown in Table 1, Olympic equestrian disciplines in Spain involve a substantial number of participating horses and events held throughout the year. Among these disciplines, Show Jumping is the most represented, with the highest number of events, participations, participating horses, and average number of participations per event. In contrast, Eventing competitions exhibit the lowest overall figures. These differences reflect the relative structural weight of each discipline within the national performance recording system.

This may be attributed to the complexity and heterogeneity of this sport, which requires different physical and mental aptitudes [14,15], the important organizational requirements of the events, and the advanced performance levels demanded from horses and riders in the three distinct phases of the discipline, which carry the greatest risks of injury and fatality for both horses and riders [16]. This competitive structure has direct implications for genetic evaluation, as disciplines with higher participation density generate more robust EBV estimates, while disciplines such as Eventing remain constrained by limited data availability and greater environmental heterogeneity.

National genetic evaluation systems vary significantly across countries due to diverse breeding objectives and testing schemes [1]. Current genetic evaluations in European countries rely on performance data from Olympic disciplines. Belgium and France use competition results; Denmark and Sweden rely on performance test results; while Germany and the Netherlands combine both competition and test results [17]. According to Ricard et al. [7], genetic progress in sport horse breeding can be accelerated when selection is based on multi-trait genetic evaluations of stallions and mares, conducted through sequential steps that integrate information from both stallion and mare tests, as well as competition data. The Spanish evaluation framework analyzed in this study follows this integrated approach, combining information from both competition and young horse performance tests.

The main breeding objective for sport horses is the talent and motivation required to achieve high performance levels and to compete successfully at that level. Therefore, competition scores for mares and stallions, as well as results from young horse competitions, must be included in routine genetic evaluations as indicator traits and early selection criteria [18]. In this context, the official equine Breeding Programs in Spain also incorporate Olympic equestrian disciplines as breeding objectives, with sufficient heritability levels (Table 2) to allow selection, taking into account both competition results and young horse competitions outcomes. In addition, other breeding objectives, such as functional conformation, behavior and/or endurance, are also included in the official Breeding Programs (Table 3) of the different riding breeds. However, the relative weight given to sport performance traits differs substantially among populations, which may influence both the intensity and effectiveness of genetic selection for Olympic disciplines.

It is remarkable that, in Spain, a unique genetic evaluation by discipline is conducted annually, encompassing all animals under performance control from different populations with an official studbook registered in the country. This procedure, which includes breed as a fixed effect and improves the genetic connectedness between the different populations, allows for the direct comparison of the various horse populations competing in Olympic disciplines based on their EBVs (additive component) under a genetic common base. This unified evaluation framework represents a distinctive feature of the Spanish breeding system, as it enables direct and objective genetic comparisons across breeds under a common methodological structure. It should be emphasized that EBV reflects the additive genetic component of performance. Although crossbred populations, such as CDE, may benefit phenotypically from heterosis effects, these correspond to non-additive genetic components (e.g., dominance or epistasis) that are not transmitted as breeding values and are therefore not captured as additive genetic merit in the BLUP animal model. Consequently, the genetic trends discussed in this study represent changes in additive genetic merit rather than heterotic effects. Notably, a high percentage of CDE horses have available EBVs for Dressage (25.27% of the official census) and Show Jumping (20.19%). Additionally, a significant proportion of PSI horses have been evaluated for Show Jumping ability (21.59%) and Eventing (20.04%), similar to the situation reported in other European populations [19]. Furthermore, 20.77% of the PRE horses included in the official census have also been genetically evaluated in 2024 for Dressage ability. These rates of genetic evaluation coverage are noteworthy and reflect the considerable sportive use of the different populations bred in Spain, the increasing interest of the sector in sport abilities, and the availability of objective and official genetic information for use in future mating design within the official Breeding Program.

To analyze genetic progress and selection intensity, only the three populations with official Breeding Programs that include Olympic disciplines were considered, as genetic efforts in these breeds are specifically focused on enhancing performance in these areas, unlike the remaining riding breeds in Spain.

The impact of breed on genetic evaluation has been widely discussed in the literature. Several authors have highlighted the influence of breed on performance data [12,20,21,22]. Ricard and Touvais [23] suggested that differences among breeds may reflect underlying genetic factors associated with variations in metabolic conditions [24,25,26], muscle morphology [27,28,29] and locomotion characteristics [30,31]. In this sense, Stewart et al. [12] recommended including the breed effect in the genetic evaluations to obtain more accurate estimates by accounting for the variance associated with breed differences. However, Doyle et al. [6] confirmed the limited number of studbooks which consider the effect of breed in their genetic models. In contrast, other studies did not detect a significant breed effect on Eventing [32] or Show Jumping performance [19]. These authors concluded that there were no significant differences between the major warmblood sport breeds produced in Europe for jumping ability. In Spain, however, the breed effect was detected as significant. Therefore, breed is included in the genetic evaluation of Olympic Disciplines (Table 2), allowing the separation of true genetic differences from population structure effects within the national evaluation system.

This study provides a unique opportunity to compare multiple equine populations under a unified national evaluation framework, because Spain implements a single genetic evaluation per discipline that integrates performance test and competition data across breeds. This shared structure may increase connectedness, enabling direct comparison of EBVs between populations, and offering valuable insight into how breed composition and Breeding Program decisions shape genetic trends under a common selection scheme.

The differences in breed participation levels across the various disciplines in Spain are evident. In Dressage, the PRE is the breed with the highest number of participations and participating horses, followed by the FWH. In Eventing, another breed with an official Spanish studbook, the AA, exhibits the highest number of participations and participating horses, followed by the FWH. In contrast, in Show Jumping competitions, the majority of registered participations and participating horses belong to the FWH, followed by CDE, both composite populations with diverse genetic backgrounds. The relatively high participation of FWH in Eventing may contribute to differences in population structure within this discipline and should therefore be considered when interpreting the genetic trends observed.

Differences in average phenotypic values are also observed across breeds (Supplementary Table S1). PRA shows the lowest scores in both Dressage and Show Jumping, similar to PSI. In these two disciplines, CDE and FWH stand out for their higher average scores. In Eventing, no breed performs consistently well across all three exercises. This pattern may be associated with the higher proportion of FWH participating in this Olympic discipline, which may enhance genetic connectedness across events but could also influence the stability of the estimated genetic trends. The highest average scores in Dressage phase are observed in FWH (although this breed shows the lowest scores in Cross-country phase), while CDE performs best in Show Jumping phase and AA in Cross-country phase. These phenotypic differences are consistent with the genetic patterns observed in EBVs and help to contextualize breed-specific genetic merit within each discipline.

From a genetic point of view, Figure 1 shows the graphical representation of the average EBV and accuracy (R) by discipline and breed, including only the three populations that feature these disciplines in their official Breeding Programs. In Dressage, the three populations analyzed exhibited broadly similar results for both the average EBV and R (Figure 1A), with CDE showing the highest R values. This may be associated with the completeness of their pedigrees (including extensive international pedigree information from imported stallions and mares) and the levels of connectedness in their participations, due to the high number of participations per animal. As affirmed by Thorén-Hellsten et al. [33], the accuracy of EBVs increases with greater connectedness among populations. In this discipline, CDE stands out due to both its composite genetic background and its high level of participations. PRE horses exhibited the highest levels of participation, although they did not achieve the highest EBV. This could be due to the positive effect of international breed promotion for sporting activities, developed by the Breeders’ Association, which increases the number of participating horses in official competitions, as well as a previously discussed breed-related differences in evaluation context [12], which may influence performance controls and official competitions results.

For Show Jumping (Figure 1B), CDE horses showed the highest levels of EBV and R compared to the other analyzed populations. This may be due to the composite nature of this population, as its official studbook allows the inclusion of breeding stock from other national and international breeds to improve sport performance ability [34] and to achieve the desired characteristics in the offspring [15]. Important selection has been made to improve Show Jumping performance in this breed with an international impact.

For Eventing (Figure 1C), the three analyzed populations exhibited similarly moderate results for the average EBVs, with AA being the population with the highest R values. This could be attributed to the higher number of registered participations, participants and participation rate per animal, which enhances the connectedness between the performance controls for this population and discipline. Overall, these results may indicate that the unified evaluation system discriminates genetic merit more effectively through accuracy and extreme-ranking animals than through differences in mean EBVs between breeds, which is consistent with expectations in well-connected multi-breed populations.

Regarding sex distribution, most of the competing horses were males across the three Olympic disciplines analyzed. However, in Dressage, geldings outnumbered females in six of the nine breed groups considered in the study (PRA, PSI, HA, FWH, Pon and Oth, Table 1), mainly because of their easier management characteristics in this strict discipline, where temperament may condition performance. In Show Jumping, the number of female CDE horses exceeded that of males and geldings, which may be explained by the high availability of mares on the market for this emerging breed in Spain as evidenced by the official census. Finally, in Eventing, females outnumbered males in five of the nine breed groups (HA, AA, FWH, Pon, and Oth), likely due to management and availability reasons in this complex discipline.

As affirmed by Viklund et al. [35], a well-functioning Breeding Program is essential to achieve defined breeding objectives, and an efficient program can only be established if past and present population conditions and trends are well characterized. In the graphical representation of the genetic progress for the three main populations selected by Olympic disciplines in Spain (Figure 2), a clear positive and significant genetic trend was observed for CDE in Dressage and Show Jumping. These results suggest an increasing specialization of the CDE populations through the selection of specific ancestors from other populations according to their EBVs and performance data, mainly in these disciplines, as evidenced by Bartolomé et al. [36]. These authors reported that, in general, EBVs of CDE offspring were higher than the EBVs of their parents, consistent with the positive trend observed in the present study.

The PRE population shows a clear positive and significant genetic tendency in Dressage. Due to its natural collection, balance and responsiveness, which suit the discipline’s precision and controlled movements, PRE horses are mainly used for Dressage competitions (Table 1). However, its conformation, characterized by a compact body [37] and limited stride length [38,39], tends to reduce scope and power, making the breed less competitive in other disciplines. The more limited response observed in Eventing is consistent with lower participation rates and greater environmental heterogeneity, which may constrain both EBV accuracy and the expected response to selection in this discipline. In this sense, both PRE and AA horses populations have shown a slight increasing trend in Eventing, with marginal significance.

Differences in genetic trends among populations therefore may reflect variation in selection intensity, generational interval and the timing of reproductive use of elite animals, rather than genetic potential alone.

Some caution is warranted when interpreting genetic trends, particularly in Eventing, where the number of observations and the unequal representation of breeds may limit the precision of the estimates. Differences in connectedness between disciplines and across years may also influence the accuracy of EBVs. Nevertheless, the consistency of results across multiple indicators (EBVs, R values and phenotypic patterns) may reinforce the robustness of the conclusions.

Reproductive technologies such as artificial insemination and embryo transfer have facilitated the dissemination of elite sport-horse genetics across countries and breeds, overcoming geographical barriers [6]. However, closed populations such as PRE and PSI remain restricted to their own genetic resources within their respective official studbooks. This limited genetic base may slow performance improvement, but enhances the genetic and economic value of purebred populations, as a genetic reservoir, providing an important potential source of genetic improvement in performance [40] and adaptability to environmental changes, and management systems.

The average EBVs and their accuracies (R) by discipline were compared among the three selected populations in order to assess genetic differences. This comparison is feasible due to the joint genetic evaluation applied across all Spanish sport horse populations. As shown in Table 4, for Dressage, the three populations exhibited similar results for TP and GGI, with CDE performing slightly better in both the complete and top 10% populations. These findings are consistent with the observed phenotypic trends. CDE also showed the highest average R values, likely attributable to a higher mean number of participations per horse, which may improve connectedness among performance records (Table 1). Together with the positive EBV trends observed for both CDE and PRE (Figure 2A), these results may support the effectiveness of the breeding programs implemented in these populations for improving Dressage ability. In contrast, AA did not stand out in terms of either EBVs or R, presenting the lowest EBVs in the top 10% population and no significant positive EBV trend (Figure 2A) which may suggest a limited response to selection for this discipline.

Part of the variability observed between breeds can also be attributed to differences in population structure and historical selection pressures. Closed populations, such as PRE, tend to display higher within-breed connectedness and more homogeneous pedigrees, which may enhance the stability of EBV estimation but may limit the introduction of novel alleles affecting performance. In contrast, composite or recently formed populations, such as CDE, benefit from broader genetic variability and introgression from performance-oriented lines, which may accelerate genetic progress but may also generate greater heterogeneity in response to selection. These structural differences may influence both the statistical properties of EBVs and the expected genetic gain across disciplines.

In Show Jumping (Table 4), CDE showed the highest EBV and R values, as expected given their strong phenotypic performance (Supplementary Table S1) and the highest number of participating horses and participations recorded for this discipline (Table 1). The pronounced positive and significant EBV trend for CDE in Show Jumping (Figure 2B) may further indicate substantial recent genetic progress, reflecting effective breeding decisions and selection policies within this studbook.

Finally, for Eventing (Table 4), CDE and AA exhibited similar results for both EBVs and R, whereas PRE showed the lowest values. The EBV trends were slightly positive for PRE and AA (Figure 2C) with marginal significance, while CDE displayed a non-significant result. These patterns highlight the need to strengthen selection strategies for Eventing in Spain, particularly in the CDE population, where recent genetic progress appears to be stagnating.

It is also important to consider that genetic trends may be influenced not only by selection decisions but also by demographic dynamics, including the number of active sires and mares, replacement rates and the degree of generational overlap within each studbook. Breeds with a broader and continuously refreshed sire base, such as CDE, may tend to show faster short-term genetic progress, whereas breeds with more conservative replacement patterns, such as PRE and AA, often may show slower but more stable trajectories. In addition, fluctuations in the availability of high-performing animals for breeding, driven by market preferences or the competitive career length of elite horses, can temporarily accelerate or decelerate genetic gain. These demographic and management factors must therefore be considered when interpreting the observed genetic trends and when designing strategies to optimize long-term selection response. These structural and demographic factors are expected to directly influence breeders’ mating decisions and the realized selection intensity, as explored through the analysis of reproductive patterns in Table 5.

To evaluate the effectiveness of the official Breeding Programs, we examined breeders’ mating decisions by analyzing the average number of offspring per stallion or mare according to their genetic merit class and the age class (Table 5). As expected, younger animals (group ≤7, considered young horses in sport competitions) show a lower number of registered offspring in comparison to older stallions and mares, with this trend being more pronounced in mares. This can be explained by the general sportive use of most of the younger animals, for performance testing, before their reproductive use in all breeds and disciplines. Only some PRE stallions registered offspring in the younger age group for Dressage. Whereas results for mares are 0 or close to 0 for all the analyzed disciplines, because their reproductive use produces their unavailability for competitions by pregnancy.

Nonetheless, reproductive patterns may also be shaped by management priorities, market demand and the economic value assigned to particular bloodlines within populations. These factors frequently lead breeders to favor animals with proven sport performance or desirable pedigrees, even when their genetic merit is not among the highest within their cohort. Consequently, the expected genetic gain may be attenuated if the most genetically superior animals are underused early in life or if selection decisions rely more heavily on reputation, phenotype or rider preference than on EBVs in the official Breeding Programs.

The highest number of registered offspring corresponds to stallions included in the top 10% by GGI value and older than twelve years. The genetic selection criteria are clearest in stallions for all the disciplines, except CDE for Dressage and AA for Show Jumping and Eventing. For the mares, also, animals with GGI > 100 and older than twelve years showed the highest number of registered offspring, except for CDE in Dressage and Eventing and AA for Eventing.

However, the relationship between genetic merit and reproductive contribution was not fully linear across breeds or disciplines. In several cases, individuals belonging to intermediate GGI classes contributed disproportionately more offspring than top 10% ranked animals, suggesting that selection decisions may not always reflect the genetic hierarchy expected under an optimal breeding strategy. This pattern indicates that breeder choice may still be influenced by subjective preferences, historical lineages or short-term sport performance, rather than by systematic use of EBVs as primary selection criteria within the official Breeding Programs.

These values demonstrate the optimal application of selection criteria both in stallions and broodmares of PRE, with a preferential use of breeding stock with GGI >100 or higher for all the Olympic Disciplines. Whereas the selection criteria applied in CDE are suboptimal in Dressage for both sexes and broodmares in Eventing. The criteria for mating design applied to the AA population in Spain may also be less aligned with genetic merit for stallions used in Show Jumping, and both stallions and broodmares in Eventing. This information is of vital importance for the Breeders Associations which have to reconsider their selective strategy within the official Breeding Program of these breeds. More training courses for breeders and greater dissemination of Breeding Stock Catalogs with genetic information are needed to ensure the efficiency of the official Breeding Programs.

These findings emphasize that the effectiveness of a Breeding Program does not rely solely on the accuracy of genetic evaluations, but also on the degree to which breeders implement selection decisions based on genetic merit. Even when EBVs provide clear differentiation between animals, inconsistent mating choices may markedly reduce the realized genetic gain in the populations. Strengthening breeder engagement with genetic information and improving the practical use of EBVs are therefore essential steps to ensure that the genetic potential estimated by the evaluation system is effectively translated into population-level progress. Overall, this study demonstrates that a unified multi-breed genetic evaluation framework may support measurable genetic progress, but its long-term effectiveness ultimately depends on the alignment between estimated genetic merit and actual breeding decisions.

## 5. Conclusions

Most equine breeds raised in Spain participate in official competitions and performance tests for the Olympic disciplines, although not all of them have yet incorporated these traits into their official Breeding Programs. The availability of harmonized performance records enables a unified genetic evaluation across breeds, allowing direct and objective comparison of genetic merit among the different populations under a common scale. This unified approach revealed marked differences between breeds in genetic merit and genetic progress, together with substantial variation in the effectiveness of their official Breeding Programs.

The analysis also revealed a selection gap in several populations, indicating that selection based on genetic merit is not fully optimized in Spain. These shortcomings may compromise long-term genetic progress and reduce the efficiency of the Breeding Programs. In contrast, some populations, such as CDE and PRE, showed a more consistent alignment between genetic merit and reproductive use of breeding stock, illustrating the potential impact of sustained and coherent selection criteria.

Since EBVs are produced annually to support breeding decisions within a unified national evaluation framework, periodic evaluations of selection responses, mating patterns and program implementation remain essential. Strengthening the integration of genetic information into breeder decision-making and ensuring proper application of the Breeding Programs will be critical to achieving sustained genetic improvement in the Olympic disciplines. The Spanish multi-breed evaluation framework described here may provide a useful reference for other countries aiming to harmonize genetic evaluations in sport horse populations.

## Figures and Tables

**Table 1:** Summary of the information available for each breed and Olympic discipline: total number of events (in brackets), number of participations and participating horses grouped by sex for performance records collected between 2004 and 2023 in Spain.

Breed	Discipline	Participations (M/F/G)	Participants (M/F/G)
PRE	Dressage	43,921 (39,828/2731/1362)	4563 (4111/326/126)
	Show Jumping	774 (497/173/104)	85 (54/18/13)
	Eventing	235 (157/45/33)	55 (37/15/3)
CDE	Dressage	5973 (3498/1614/861)	574 (329/177/68)
	Show Jumping	89,091 (40,710/39,293/9088)	3422 (1413/1606/403)
	Eventing	5296 (1943/2393/960)	568 (259/243/66)
PRA	Dressage	286 (163/50/73)	30 (19/4/7)
	Show Jumping	264 (189/36/39)	46 (24/15/7)
	Eventing	667 (398/148/121)	102 (68/17/17)
PSI	Dressage	113 (39/21/53)	18 (7/5/6)
	Show Jumping	631 (324/202/105)	74 (37/21/16)
	Eventing	651 (214/223/214)	84 (36/27/21)
HA	Dressage	186 (133/7/46)	34 (25/2/7)
	Show Jumping	120 (36/70/14)	20 (10/6/4)
	Eventing	214 (65/107/42)	30 (12/14/4)
AA	Dressage	159 (57/21/81)	24 (12/7/5)
	Show Jumping	4399 (2127/1752/520)	351 (163/147/41)
	Eventing	5109 (2042/2446/621)	443 (189/212/42)
FWH	Dressage	39,503 (18,168/6529/14,806)	2723 (1287/487/949)
	Show Jumping	202,915 (100,053/70,846/32,016)	6772 (3091/2455/1226)
	Eventing	3108 (828/1183/1097)	341 (112/136/93)
Pony breeds	Dressage	1668 (473/321/874)	119 (40/30/49)
	Show Jumping	0	0
	Eventing	258 (15/135/108)	26 (4/12/10)
Others	Dressage	9284 (4635/1676/2973)	988 (518/176/294)
	Show Jumping	20,806 (10,080/7745/2981)	1339 (597/532/210)
	Eventing	1997 (629/887/481)	337 (122/137/78)
TOTAL	Dressage	101,093 (66,994/12,970/21,129)	9073 (6348/1214/1511)
	Show Jumping	319,000 (154,016/120,117/44,867)	12,109 (5389/4800/1920)
	Eventing	17,535 (6291/7567/3677)	1986 (839/813/334)

PRE is Pura Raza Española, CDE is Caballo de Deporte Española, PRA is Spanish Pura Raza Árabe, PSI is Pura Sangre Inglés, HA is Spanish Hispano-Árabe, AA is Spanish Anglo-Árabe, FWH is Foreign Warmblood Horses, M is male (not castrated), F is female and G is gelding (castrated).

**Table 2:** Description of the models utilized for the genetic evaluation of each Olympic discipline in Spain, using the 2024 genetic evaluation as a reference (performance data from 2004 to 2023).

Disc	Analyzed Traits	Trait	h^2^ (s.e.)	GGI (%)	Method	Model (Fixed and Random Effects)	Ped Size
Dressage	Walk	W	0.14 (0.027)	10	Multiple trait BLUP animal model	Fixed effects: Age (linear covariate) Breed (24) Sex (3) Event (1467) Level (11) Stud/owner (2411) Random effects: Animal (additive genetic effect; a~N(0, Aσ^2^_a_)) Rider PEE	35,589
	Trot	T	0.18 (0.031)	10			
	Canter	C	0.18 (0.031)	5			
	Submission	S	0.17 (0.027)	2.5			
	Perspective	P	0.18 (0.032)	2.5			
	Total points/reprise	TP	0.1 (0.025)	70	Univariate BLUP animal model		
Show jumping	Positive score of Jumping (200-total penalty)	PSJ	0.04 (0.006)	50	Bivariate BLUP animal model	Fixed effects: Age (linear covariate) Breed (19) Sex (3) Event (1992) Competition type (11) Level (223) Random effects: Animal (additive genetic effect; a~N(0, Aσ^2^_a_)) Animal–rider interaction (32,072) PEE	33,935
	Weighted final ranking	WFR	0.10 (0.006)	50			
Eventing	Positive score of Dressage (150-total penalty)	EDr	0.19 (0.010)	35	Multiple trait BLUP animal model	Fixed effects: Age (linear covariate) Breed (11) Sex (3) Event (640) Level (8) Random effects: Animal (additive genetic effect; a~N(0, Aσ^2^_a_)) Rider PEE	12,102
	Positive score of Jumping (125-total penalty)	ESJ	0.16 (0.011)	25			
	Positive score of cross-country (200-total penalty)	ECr	0.09 (0.009)	40			

Disc = discipline; h^2^ = heritability; GGI = Genetic Global Index (% weighting); Method = statistical approach applied (see Section 2.2); Ped size = number of animals in the pedigree file; PEE = permanent environmental effect (random); WFR = weighted final ranking (100 assigned to the highest score and 0 to the lowest). Maximum penalty values were 200 (Show Jumping), 150 (Eventing Dressage phase), 125 (Eventing Jumping phase) and 200 (Eventing Cross-country phase).

**Table 3:** Main information on the most important horse breeds bred in Spain and the orientation of their official Breeding Programs, ordered by the census reported in Spain at the end of 2024.

Breed	Census	Breeding Program	Animals with EBV		
			Dr	SJ	Ev
Pura Raza Española (PRE)	211,424	Functional conformation Behavior Classic Dressage	43,914	1806	2310
Caballo de Deporte Español (CDE)	23,513	Eventing Endurance Show Jumping Classic Dressage	5942	4747	970
Spanish Pura Raza Árabe (PRA)	18,995	Functional conformation Endurance	286	674	1490
Spanish Hispano-Árabe (HA)	12,096	Conformation and basic gaits	188	169	54
Pura Sangre Inglés (PSI)	9975	Speed races	113	2154	1999
Spanish Anglo-Árabe (AA)	9644	Functional conformation Eventing Endurance Show Jumping Classic Dressage Cowboy Dressage	159	1293	1485

EBV is estimated breeding values; Dr is dressage; SJ is Show Jumping; and Ev is Eventing.

**Table 4:** Comparison of mean EBVs between the complete population and the elite group (top 10%) and mean accuracy (R) for sport-performance traits in PRE, CDE and AA populations across the three Olympic disciplines.

		Population	Complete			Top 10%	
Disc.	Trait	Breed	N	EBV	R	N	EBV
Dressage	TP	PRE	7833	107.75	0.24	783	111.75
		CDE	515	108.38	0.41	52	112.68
		AA	18	108.11	0.32	2	110.97
	GGI	PRE	7833	106.39	0.28	783	111.20
		CDE	515	106.50	0.41	52	111.65
		AA	18	106.28	0.32	2	108.88
Show Jumping	PSJ	PRE	54	101.02	0.35	5	102.50
		CDE	2299	102.08	0.58	230	104.79
		AA	184	101.23	0.44	18	103.12
	WFR	PRE	54	101.02	0.52	5	106.12
		CDE	2299	103.46	0.72	230	109.27
		AA	184	102.86	0.62	18	107.33
	GGI	PRE	54	101.62	0.43	5	104.31
		CDE	2299	102.77	0.65	230	107.03
		AA	184	102.04	0.53	18	105.23
Eventing	EDr	PRE	133	99.23	0.43	13	105.08
		CDE	643	101.16	0.69	64	111.53
		AA	529	101.29	0.74	52	110.30
	ESJ	PRE	133	100.13	0.41	13	106.78
		CDE	643	101.14	0.68	64	110.11
		AA	529	101.85	0.72	52	110.14
	ECr	PRE	133	100.43	0.36	13	107.87
		CDE	643	100.90	0.60	64	111.05
		AA	529	101.87	0.65	52	110.68
	GGI	PRE	133	99.89	0.40	13	105.84
		CDE	643	101.03	0.65	64	110.24
		AA	529	101.65	0.70	52	109.73

Disc is discipline; PRE is Pura Raza Española, CDE is Caballo de Deporte Español, AA is Spanish Anglo-Árabe, EBV is estimated breeding value, R is accuracy, TP is total points/reprise in Dressage, PSJ is positive score of Jumping, WFR is weighted final ranking, EDr is positive score of dressage phase of Eventing, ESJ is positive score of jumping phase of Eventing, ECr is positive score of cross-country phase of Eventing, and GGI is Genetic Global Index.

**Table 5:** Average number of offspring per stallion or mare according to their Genetic Global Index (GGI) value and the age group of the breeding stock.

Sex	Age	GGI Level	Dressage			Show Jumping			Eventing		
			PRE	CDE	AA	PRE	CDE	AA	PRE	CDE	AA
Stallion	≤7	≤100	1.16	0	0	0	0	1	0	0	0.20
		>100	1.15	0	0	0	0.13	0	0	0.06	0.06
		Upper 10%	1.62	0	0	0	0	0	0	0	0.20
	>7–≤12	≤100	3.40	0.95	0	0.50	0	2	4.66	0.03	0.57
		>100	7.40	0.07	0	9.38	0.30	0.98	12.20	0.37	1.04
		Upper 10%	7.71	0.07	0	0.25	0.11	0.98	12.20	0	1.67
	>12	≤100	14.10	1.25	0	11.33	0.26	8	40.53	0.26	8.04
		>100	20.10	0.47	0.88	44.33	3.40	7.45	45.64	4.50	7.11
		Upper 10%	26.71	0.26	2.33	58.62	4.74	5.15	66.22	5.98	4.19
Mare	≤7	≤100	0	0	0	0	0	0	0	0	0
		>100	0.08	0	0	0	0	0	0	0	0.05
		Upper 10%	0.09	0	0	0	0	0	0	0	0
	>7–≤12	≤100	2.38	0	0	0	0	0	0	0.09	0.32
		>100	2.44	0.13	0	1	0.15	0.70	0	0.21	0.54
		Upper 10%	2.26	0.08	0	0	0	1.21	0	0	0.38
	>12	≤100	6.08	0.67	0	1.66	1	2.70	7.05	3.11	2.91
		>100	6.25	0.26	0.2	7.35	2.78	2.66	7.38	2.56	2.35
		Upper 10%	6.62	0.42	0	5.87	1.66	3.75	8.29	0.61	3.67

PRE is Pura Raza Española, CDE is Caballo de Deporte Español, AA is Spanish Anglo-Árabe.

## Data Availability

The datasets generated and/or analyzed during the current study are available in the Zenodo repository, https://doi.org/10.5281/zenodo.18388896. Data were anonymized and do not contain information that enables individual animal identification.

## Supplementary Materials

The following supporting information can be downloaded at: https://www.mdpi.com/article/10.3390/life16030455/s1, Table S1: Average phenotypic scores by breed for traits included in the genetic evaluation of Olympic disciplines in Spain.
