# Supplementary material for: Integrating Performance Records and Genetic Evaluations in Spanish Horse Populations Competing in Olympic Disciplines
Source: Life (Basel). 2026 Mar 10;16(3):455. doi: 10.3390/life16030455 (PMC13028280; doi:10.3390/life16030455)
Supplement: Supplementary file 1 [file life-16-00455-s001.zip › life-4151341-supplementary.pdf]

Table S1. Average phenotypic scores by breed for traits included in the genetic evaluation of Olympic disciplines in Spain.

Discipline	Dressage						Show Jumping		Eventing		
**Trait/Breed**	**W**	**T**	**C**	**S**	**P**	**TP**	**PSJ**	**WFR**	**EDr**	**ESJ**	**ECr**
**Pura Raza Español (PRE)**	6.70	6.79	6.85	6.62	6.77	65.94	191.47	63.09	102.73	117.51	189.41
**Caballo de Deporte Español (CDE)**	6.96	6.88	6.93	6.65	6.87	66.06	191.67	65.39	102.66	119.33	186.01
**Spanish Pura Raza Árabe (PRA)**	6.02	6.23	6.13	6.00	6.12	61.94	189.41	64.11	104.18	117.96	187.82
**Pura Sangre Inglés (PSI)**	6.52	6.58	6.43	6.18	6.49	63.07	189.90	52.10	102.47	115.90	185.99
**Spanish Hispano-Árabe (HA)**	6.53	6.54	6.49	6.36	6.48	64.79	189.72	53.15	102.28	118.91	189.18
**Spanish Anglo-Arabe (AA)**	6.58	6.30	6.15	5.99	6.24	63.45	191.27	64.22	104.38	119.30	189.62
**Foreign Warmblood Horses (FWH)**	7.26	7.27	7.26	6.89	7.20	66.55	191.85	67.36	106.53	119.03	183.81

Where W is walk, T is trot, C is canter, S is submission, P is perspective, TP is total points/reprise, PSJ is Positive score of Jumping, WFR is weighted final ranking, EDr is positive score of dressage phase of Eventing, ESJ is positive score of jumping phase of Eventing, ECr is positive score of cross country phase of Eventing.
